# Defining concepts in cancer survivorship

**DOI:** 10.1002/cncr.70039

**Published:** 2025-08-14

**Authors:** Michelle A. Mollica, Michelle Doose, Crystal Reed, Emily Tonorezos

**Affiliations:** ^1^ Division of Cancer Control and Population Sciences Office of Cancer Survivorship National Cancer Institute Bethesda Maryland USA

**Keywords:** cancer survivors, caregivers, definitions, survivors, survivorship

## Abstract

The National Cancer Institute’s Office of Cancer Survivorship (OCS) leads efforts to define and address the complex and evolving needs of cancer survivors, caregivers, and families through research, care, collaboration, and advocacy. This article provides updated definitions for key terms—cancer survivor, survivorship, survivorship care, and survivorship research—to guide practice, policy, and research in improving outcomes across the cancer care continuum.

The National Cancer Institute’s Office of Cancer Survivorship (OCS) was established in 1996 in response to a call from survivor advocates, in recognition of the growing number of people surviving cancer for long periods of time and their poorly understood needs. The mission of OCS is to support research that examines and addresses the long‐ and short‐term physical, psychological, social, and economic effects of cancer and its treatment among survivors of all ages and stages and their caregivers and families.^1^ This work is accomplished by 1) advancing scientific research; 2) supporting investigators and training to foster a robust network of extramural cancer survivorship research leaders; 3) communicating and collaborating with internal and external survivorship experts; and 4) promoting continued growth and sustained improvement in cancer survivorship care.

Survivor advocates, who saw the creation of OCS as a culmination of their efforts, also started a movement in calling themselves cancer survivors. Concurrently, researchers began to learn more about concerns and needs related to treatment and survivorship. For cancer survivors, advocates, and researchers, OCS has and continues to serve as the lead in developing a taxonomy of key concepts related to cancer survivorship and caregiving. The purpose of this article is to provide an update on common definitional terms, including cancer survivor, cancer survivorship, cancer survivorship care, and cancer survivorship research. These definitions were developed through a consensus process that included a comprehensive review of the literature and meetings with survivor advocates and subject matter experts.

## CANCER SURVIVOR

**FIGURE 1:**
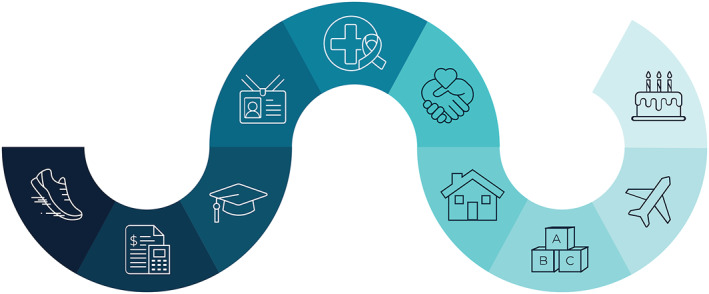
Definition of cancer survivor: a cancer survivor is anyone from the time of diagnosis throughout the balance of life. This figure represents that cancer occurs alongside the many events within a person’s life.

An individual is considered a cancer survivor from the time of diagnosis through the balance of life,^2^, ^3^ and it is recognized that people experience a cancer diagnosis alongside the many events and phases of their lives (Figure 1). There are many types of survivors, including those living with cancer and those free of cancer. This term is meant to encompass the population with a history of cancer rather than to assign an individual label that may or may not resonate with all survivors. Although each individual’s experience as a survivor is unique, there are different types of cancer survivors, including those who are diagnosed with early‐stage cancer, those who are diagnosed with advanced or metastatic cancer or who progress to metastatic cancer, and those who are diagnosed with or progress to end‐stage cancer.^4^ Goals of care for survivors, including being treated for curative intent or to maximize quality of life, may vary based on several factors, including cancer type, treatment(s) received, age, and prognosis.

## CANCER SURVIVORSHIP

**FIGURE 2:**
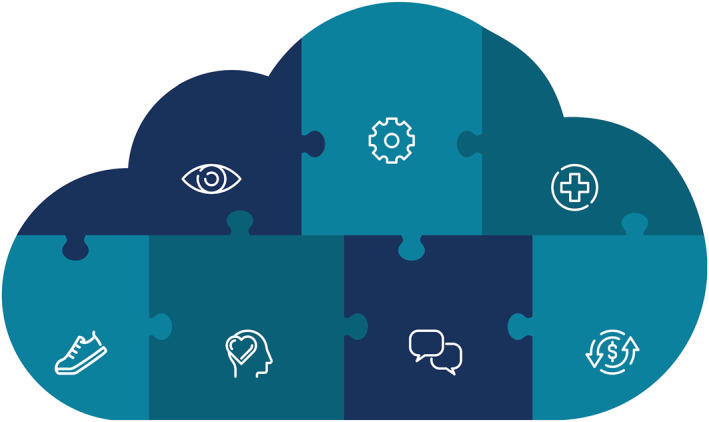
Definition of cancer survivorship: cancer survivorship is a complex state of being. It includes the perspectives, needs, health, and the physical, psychological, social, and economic challenges experienced by people and caregivers after a cancer diagnosis.

Cancer survivorship is a complex state of being. It includes the perspectives, needs, health, and the physical, psychological, social, and economic challenges experienced by survivors and caregivers after a cancer diagnosis (Figure 2).^2^

## CANCER SURVIVORSHIP CARE

**FIGURE 3:**
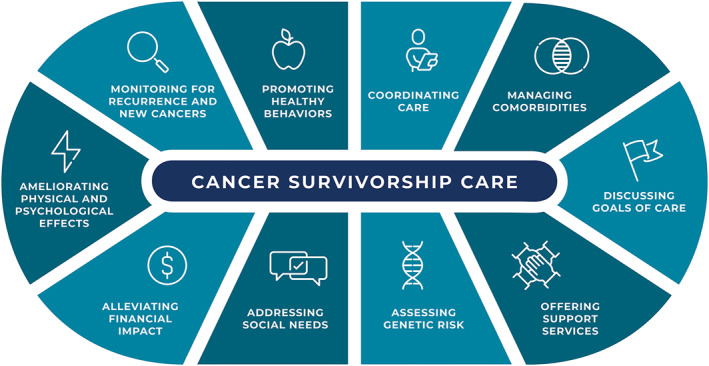
Definition of cancer survivorship care: cancer survivorship care is comprehensive care for all people with a history of cancer and begins at the time of diagnosis, with the goal of assessing and mitigating the impact of cancer and its treatment.

Care for a person after a cancer diagnosis is termed cancer survivorship care. It is comprehensive care for all people with a history of cancer and begins at the time of diagnosis (Figure 3).^2^, ^5^ The goal of cancer survivorship care is to assess and mitigate the impact of cancer and its treatment, and it includes several components of care:

- Surveillance and amelioration of physical, emotional, and psychological effects, including evaluation of risk, prevention, and management of long‐term (continues for months or years) and late effects (diagnosed months or years after treatment);
- Surveillance for recurrence and new cancers;
- Assessment and promotion of health behaviors (e.g., tobacco use cessation, physical activity, alcohol use cessation, weight management);
- Coordination of care between care team members, health systems, survivors, and caregivers;
- Addressing comorbidities and preventing and managing chronic conditions exacerbated by cancer and its treatment;
- Engagement in care planning, including discussing goals of care and advanced care planning;
- Provision of supportive health services (e.g., nutrition, occupational and physical therapy, rehabilitation, sexual health, fertility services, dental, and podiatry services);
- Genetic risk assessment or referral to genetic testing as appropriate;
- Management of social risks, health‐related social needs, education and employment; and
- Addressing financial hardship and insurance coverage and the provision of related services.

This care is often delivered by multiple types of health care providers, with a goal of providing comprehensive, needs‐based care to people after a cancer diagnosis.

## CANCER SURVIVORSHIP RESEARCH

**FIGURE 4:**
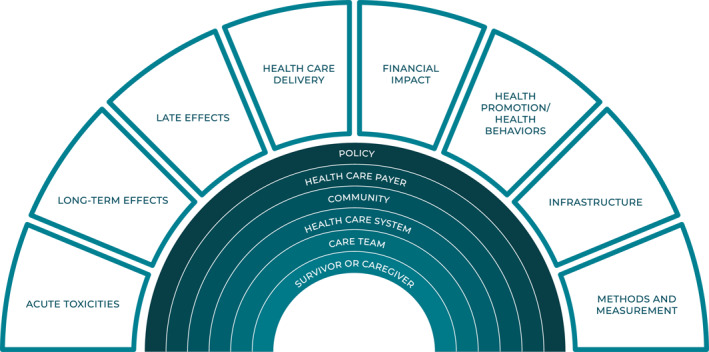
Definition of cancer survivorship research: cancer survivorship research aims to understand, prevent, and mitigate acute and late‐occurring physical, psychological, social, and economic effects of cancer and its treatment, improve care delivery, promote healthy behaviors, develop and sustain infrastructure, and improve research methodologies for individuals impacted by cancer.

Research focused on cancer survivorship aims to enhance the health and well‐being of all cancer survivors and caregivers (Figure 4).^2^ This research area aims to understand, prevent, and mitigate acute and late‐occurring physical, psychological, social, and economic effects of cancer and its treatment, improve care delivery, promote healthy behaviors (e.g., diet and physical activity, smoking cessation, and preventive care), develop and sustain research infrastructure (e.g., data repositories, research hubs, and biospecimen collection), and improve research methodologies for individuals impacted by cancer. Additionally, research on the multilevel contextual factors that influence aspects of cancer survivorship, including factors related to health policy, health care payer, community, health care system, care team, and survivor and caregiver characteristics (e.g., socioeconomic status, sex, and race/ethnicity), are included in the definition of cancer survivorship research.

## CONCLUSION

With over 18 million cancer survivors in the United States,^6^ we recognize that the terms survivor and survivorship may not resonate with all people with a history of cancer. Instead, these definitions are meant to capture a population or associated phenomena that can be used by survivor advocates and organizations to inform research, clinical care, and health policy. It is likely that these terms and their meanings will evolve over time with advances in our understanding of the needs and perspectives of cancer survivors.

## AUTHOR CONTRIBUTIONS

**Michelle A. Mollica**: Conceptualization; writing—original draft; writing—review and editing. **Michelle Doose**: Writing—review and editing. **Crystal Reed**: Writing—review and editing. **Emily Tonorezos**: Conceptualization; writing—review and editing.

## CONFLICT OF INTEREST STATEMENT

Emily Tonorezos reports fees for professional activities from Weill Cornell Medicine. The other authors declare no conflicts of interest.

## Data Availability

Data sharing not applicable to this article as no data sets were generated or analyzed during the current study.
